# The Gut-Brain-Microbiome Axis and Its Link to Autism: Emerging Insights and the Potential of Zebrafish Models

**DOI:** 10.3389/fcell.2021.662916

**Published:** 2021-04-15

**Authors:** David M. James, Elizabeth A. Davidson, Julio Yanes, Baharak Moshiree, Julia E. Dallman

**Affiliations:** ^1^Department of Biology, University of Miami, Coral Gables, FL, United States; ^2^Department of Gastroenterology and Hepatology, Atrium Health, Charlotte, NC, United States

**Keywords:** gastrointestinal, microbiome, comorbidities, ASD, GI, CNS

## Abstract

Research involving autism spectrum disorder (ASD) most frequently focuses on its key diagnostic criteria: restricted interests and repetitive behaviors, altered sensory perception, and communication impairments. These core criteria, however, are often accompanied by numerous comorbidities, many of which result in severe negative impacts on quality of life, including seizures, epilepsy, sleep disturbance, hypotonia, and GI distress. While ASD is a clinically heterogeneous disorder, gastrointestinal (GI) distress is among the most prevalent co-occurring symptom complex, manifesting in upward of 70% of all individuals with ASD. Consistent with this high prevalence, over a dozen family foundations that represent genetically distinct, molecularly defined forms of ASD have identified GI symptoms as an understudied area with significant negative impacts on quality of life for both individuals and their caregivers. Moreover, GI symptoms are also correlated with more pronounced irritability, social withdrawal, stereotypy, hyperactivity, and sleep disturbances, suggesting that they may exacerbate the defining behavioral symptoms of ASD. Despite these facts (and to the detriment of the community), GI distress remains largely unaddressed by ASD research and is frequently regarded as a symptomatic outcome rather than a potential contributory factor to the behavioral symptoms. Allowing for examination of both ASD’s impact on the central nervous system (CNS) as well as its impact on the GI tract and the associated microbiome, the zebrafish has recently emerged as a powerful tool to study ASD. This is in no small part due to the advantages zebrafish present as a model system: their precocious development, their small transparent larval form, and their parallels with humans in genetics and physiology. While ASD research centered on the CNS has leveraged these advantages, there has been a critical lack of GI-centric ASD research in zebrafish models, making a holistic view of the gut-brain-microbiome axis incomplete. Similarly, high-throughput ASD drug screens have recently been developed but primarily focus on CNS and behavioral impacts while potential GI impacts have not been investigated. In this review, we aim to explore the great promise of the zebrafish model for elucidating the roles of the gut-brain-microbiome axis in ASD.

## Introduction

**FIGURE 1:**
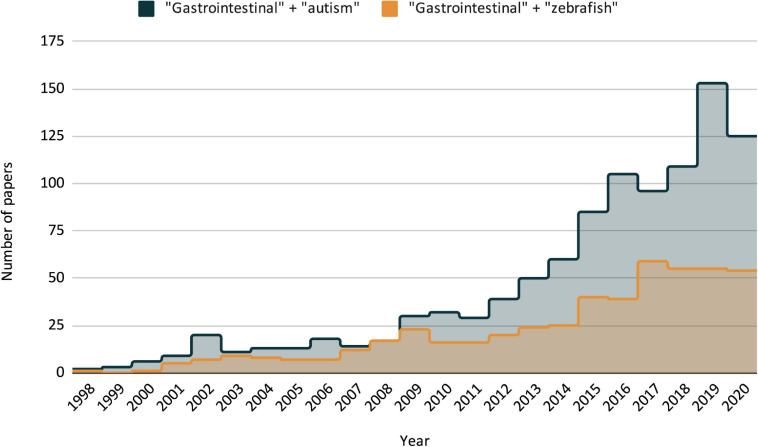
Publication trends as listed by PubMed/NLM over the last two decades, search criteria for each is “gastrointestinal + autism” and “gastrointestinal + zebrafish.” Including all three search terms (GI + ASD + zebrafish) only resulted in three publications, the earliest in 2014.

The contribution of the gut-brain-microbiome axis to health and disease states is a relatively new field of research (Figure 1) with increasing interest from both public and scientific spheres (Drossman and Hasler, 2016). An understanding of this axis draws on a range of disciplines including neurobiology, gastroenterology, microbiology, endocrinology, and psychology (Liang et al., 2018; Neuhaus et al., 2018). This breadth of subjects relevant to the gut-brain-microbiome field speaks to the diversity of its potential applications. Specifically, its relevance to research on neurological disorders like autism spectrum disorder (ASD) is of particular interest, as such disorders often cause a wide range of symptoms involving multiple body systems. Furthermore, the interconnected aspects of the gut-brain-microbiome offer alternative causal explanations and treatment strategies for symptoms traditionally understood to be strictly caused by deficits within the central nervous system (CNS) (Neuhaus et al., 2018; Lefter et al., 2019; Srikantha and Mohajeri, 2019; Tye et al., 2019). While ASD is still diagnosed by deficits in social communication, repetitive behaviors, and/or restrictive interests, comorbidities (co-occurring symptoms) like seizures, epilepsy, sleep disturbance, hypotonia, and GI distress are also common with significant negative impacts on quality of life (Christensen et al., 2018; “IACC, 2019 Strategic Plan For Autism Spectrum Disorder 2018–2019 Update,” 2019; Leader et al., 2020). Here, we review how recent studies of the gut-brain-microbiome axis have changed our understanding of ASD related symptoms and highlight the important role the zebrafish model can play in future research.

Since it was first described in a small subset of patients in 1943, the clinical definition of ASD has been subject to an ever-changing set of criteria in an attempt to capture a condition that is both common and heterogeneous. Likewise, estimates of the prevalence of comorbidities associated with ASD have changed (Geschwind, 2009; Chaidez et al., 2014; Bresnahan et al., 2015; De Rubeis and Buxbaum, 2015; Tye et al., 2019). This shift in criteria likely stems from diverse causal factors including hundreds of implicated genes, environmental, and gene-environment interactions that contribute to ASD prevalence (Chaste and Leboyer, 2012). Our current understanding is that ASD impacts more than 1% of the population and is both etiologically and clinically heterogeneous. Given this heterogeneity, addressing underlying mechanisms to develop treatment strategies has been difficult (Manoli and State, 2021). Moreover, the field would greatly benefit from determining how body systems work cooperatively and/or antagonistically to produce both core behavioral symptoms of ASD and the comorbid symptoms like gastrointestinal (GI) distress (McElhanon et al., 2014; Pellicano et al., 2014; Frye et al., 2015; Latorre et al., 2016; Rao and Gershon, 2016; Rose et al., 2017; Goodspeed et al., 2020). GI distress occurs at a disproportionately higher rate in individuals with ASD than the general population, and symptom severity ranges from relatively low-impact to severe (Bresnahan et al., 2015). As research into these GI symptoms has expanded, mounting evidence suggests that they also contribute to the behavioral symptoms associated with ASD, a finding well-recognized even in patients without ASD and explained by the biopsychosocial model of disorders of gut-brain interaction (Klarer et al., 2014; Mayer et al., 2014; Drossman and Hasler, 2016; Sharon et al., 2019). With this in mind, it becomes apparent why the gut-brain-microbiome axis is a critical focal point for studying both the pathophysiology of ASD-related GI dysfunction and ASD as a whole.

Addressing GI symptoms within neurological disorders is challenging because the regulation of GI function is complex and full of redundant feedback mechanisms involving multiple body systems (Holtmann and Talley, 2014). Adding to this complexity is the fact that the luminal space of the GI tract is technically “outside” of the body and not sterile, lending itself to microbial and chemical exposure which could influence regulatory mechanisms. Under normal conditions, communication between the GI tract and the CNS is modified by contributions from immune, microbial, hormonal, motor, and sensory inputs (Grundy et al., 2006; Vanner et al., 2016). The GI tract also exerts a large amount of autonomous control over its own functions, with the enteric nervous system (ENS) interfacing with various mechanosensory, chemosensory, endocrine, immune, and secretory cells, altering GI function as needed to deal with threats and maintain homeostasis (Holtmann and Talley, 2014). These typical functions may be altered in ASD (Hsiao, 2014), and recent findings from both clinical and rodent model studies have begun to frame the importance of the gut-brain-microbiome axis in ASD (Hsiao, 2014).

Viewing autism as a disorder of the brain, without consideration of gut/microbiome can have unintended negative consequences; a prime example is the use of antipsychotics to reduce aggressive behaviors, since these also suppress GI motility and thus are likely to increase GI distress (de Alvarenga et al., 2017). In this review, we contend that the zebrafish presents unique opportunities to approach to autism research holistically. In particular zebrafish ASD models are amenable to genetic modification, *in vivo* visualization of multiple organ systems, and high-throughput studies, providing an ideal model system to address a multidisciplinary gut-brain-microbiome approach to ASD research (Brugman, 2016; Kozol et al., 2016; Phelps et al., 2017; Kozol, 2018; James et al., 2019).

## Gastrointestinal Issues and Their Link to Neurological Disorders

Gastrointestinal distress is a pervasive co-occurring ailment in a wide range of neurological disorders, including Parkinson’s (Mulak and Bonaz, 2015; Liddle, 2018; Brudek, 2019), schizophrenia (Severance et al., 2015, 2016; Dickerson et al., 2017), and Alzheimer’s (Hill et al., 2014; Jiang et al., 2017; Kowalski and Mulak, 2019; Goyal et al., 2020; He et al., 2020). Only in the last 5 years has GI distress been more widely recognized as an ASD-related comorbidity, and the potential causes have been the subject of considerable and ongoing debate. Although a comprehensive discussion on the clinical prevalence and significance of GI distress in ASD is outside of the scope of this review, we believe reviewing a few critical points are helpful in framing the current state of zebrafish-based research as it relates to ASD and GI comorbidities.

Broadly speaking, the link between psychological and gastrointestinal states has been acknowledged for centuries (Wolf, 1981), though this understanding has not been applied to neurodevelopmental disorders like ASD until recently. In fact, while research from the late 90s and early 2000s explored links between ASD and GI distress, no thorough categorization or treatment of ASD-related GI distress was attempted until 2010 (Buie et al., 2010). This interdisciplinary panel was unable to link a specific GI pathophysiology to individuals with ASD; nonetheless, they agreed that the prevalence of GI abnormalities was not completely understood, GI symptoms are frequently linked with negative behavioral manifestations, and that more research was required before coming to any definitive conclusions on evidence-based treatment recommendations. Current clinical research into the ASD-GI link is still hindered by many of the same obstacles that were identified over a decade ago: inconsistent or varying criteria used to define GI phenotypes, inconsistent or varying methodology (including differences in the reporting and measuring of GI phenotypes), and inconsistent criteria for patient participation and selection. This explains, in part, the wide variation in reported prevalence of ASD related GI symptoms, which ranges from 23% to 70% (Chaidez et al., 2014; McElhanon et al., 2014). Recent work has attempted to address these issues (Bresnahan et al., 2015). In a prospective population-based cohort study with well-defined methodology and participation criteria, Bresnahan et al. (2015) has shown that individuals with ASD are not only more likely to experience GI-related problems when compared to their typically developing counterparts, but that the type of GI distress varies with age. Encompassing a 10-year period, 95,278 mothers (with 114,516 children) from the Norwegian Mother and Child Cohort Study (MoBa) were recruited to participate, with “ongoing follow-up [including] health, behavioral, developmental, and nutritional questionnaires and collection of clinical and biological data” and maternal reports of GI symptoms. Additionally, by simplifying the categories of GI distress to only include constipation, food allergy, and diarrhea, the study focused on easily identifiable symptoms and limited the possibility of over or underreporting. This is a particularly important consideration when dealing with children who have communication deficits, or with non-verbal autistic individuals, irrespective of age. This study represents the first large-scale prospective cohort study on ASD-related GI symptoms that confirms GI distress existing at a higher rate within the ASD population. It also underscores the need for not only more GI-related ASD research, but for unified and consistent approaches to measuring GI distress (Margolis et al., 2019).

**FIGURE 2:**
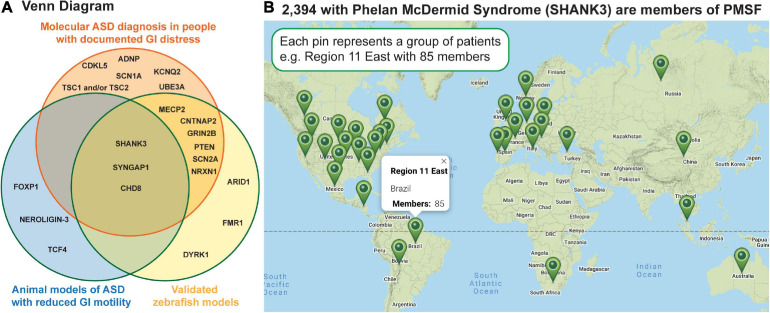
Human genetics has identified 100s of sporadic, *de novo* genetic changes that can cause ASD; shown are a subset of these that report GI distress as a major symptom. **(A)** The Venn diagram shows genes linked to GI distress in ASD in the orange circle, those which have extant zebrafish models in the blue circle, and those in which reduced GI motility has been reported in an animal model. **(B)** The map shows where families caring for individuals with Phelan McDermid Syndrome are scattered across the globe making a standard of care challenging. This Google map image was generated by the Phelan McDermid Syndrome Foundation and is reproduced above with their permission.

In addition to prospective studies, severe GI symptoms have been reported in at least eighteen molecularly identified forms of ASD (Table 1), many of which have corresponding zebrafish and/or rodent models that exhibit reduced intestinal tract motility (Figure 2). Because many of these molecularly identified forms of ASD are rare, caused by sporadic *de novo* genomic changes, clinical needs of these individuals can often go unmet (*SHANK3*; Figure 2B). Interestingly, gene expression analyses have shown that many of these ASD-linked genes are expressed in the intestine in both mammals (Sauer et al., 2019) and zebrafish (Lavergne et al., 2020; Wen et al., 2020; Willms et al., 2020) raising the possibility that GI distress is caused by gut-intrinsic mechanisms. Because GI homeostasis is maintained through a concert of influence from the CNS, the microbiome, and the GI tract itself, studies focusing on how the three interact stand to provide the most comprehensive explanations for why GI distress is prevalent within ASD populations.

**TABLE 1:** Molecularly-defined forms of ASD with GI symptoms.

Genetic locus	*Foundation:* Reported GI distress	Publications reporting GI distress case reports^*CR*^
***ADNP***	*ADNP Kids Research Foundation:* GERD, reflux, cyclical vomiting, constipation, diarrhea, delayed digestion, stomach ulcers/scarring, IBS	Van Dijck et al. (2019)
***CDKL5***	*International Foundation for CDKL5 Research:* Abdominal distension, constipation, diarrhea, reflux, slow gastric emptying, low motility, risk of life-threatening volvulus and intussusception	Amin et al. (2017)
***CHD8***	*SPARK:* Gastrointestinal issues	Bernier et al. (2014)
***CNTNAP2***	*Pitt Hopkins Research Foundation*: Syndrome 1 constipation	Gregor et al. (2011)
***Dup Chr 15q***	*Dup 15 Q Alliance:* Feeding issues in infancy, encopresis, acid reflux, some with G-tube	Shaaya et al. (2015)
***FOXG1***	*International FOXG1 Foundation:* Constipation	McMahon et al. (2015)
**FOXP1**	Siper et al., 2017 found that of 9 people with *FOXP1* syndrome, 3 had feeding issues and 4 had constipation.	Frohlich et al. (2016, 2019), Siper et al. (2017)
***KCNQ2***	*KCNQ2 Cure Alliance:* GI issues seen commonly	Inagaki et al. (2019)
***MECP2***	*Rett syndrome/Rett Syndrome Research Trust:* 92% prevalence of GI dysmotility	Motil et al. (2012)
***NRXN1***	*Pitt Hopkins Research Foundation*: Syndrome 2 constipation, reflux	Zweier et al. (2009); Harrison et al. (2011)
***PTEN***	*PTEN Hamartoma Tumor Syndrome Foundation:* Intestinal hamartomatous polyposis	Shaco-Levy et al. (2017)
***SCN1A***	*Dravet Syndrome Foundation:* Constipation, dysmotility	Villas et al. (2017)
***SCN2A***	*Families SCN2A Foundation:* Reflux and constipation	Tian et al. (2019)
***SHANK3***	*Phelan McDermid Syndrome Foundation:* Constipation, reflux, some with G-tube	De Rubeis et al. (2018)
***SYNGAP1***	*Bridge the Gap SYNGAP1 ERF:* Constipation, reflux, links btn GI and aggression, some with G-tube.	Parker et al. (2015); Prchalova et al. (2017)
***TCF4***	*Pitt Hopkins Research Foundation:* Gastrointestinal issues	Peippo and Ignatius (2012)
***TSC 1 and 2***	*Tuberous Sclerosis Alliance:* Rectal bleeding, papillomas in GI tract, constipation	Moulis et al. (1992)
***UBE3A***	*Angelman Syndrome Foundation Inc.:* Constipation/possibly due to low truncal tone, reflux/gagging	Williams et al. (2010)

## The Role of the CNS in the Gut-Brain-Microbiome Axis

Gastrointestinal function is regulated by crosstalk between the nervous system, the gut, and the microbiome (Stengel and Tache, 2010; Drossman and Hasler, 2016; Zhao and Pack, 2017; Ganz, 2018) and, as such, disruptions to this cross talk can contribute not only to GI distress (Bielefeldt et al., 2016) but also to core behaviors used to diagnose ASD (Chaidez et al., 2014; McCue et al., 2017; Penzol et al., 2019). The brain tracks gut luminal contents via sensory enteroendocrine cells (EECs) scattered throughout the gut lining. EECs signal using hormones like serotonin and cholecystokinin (CCK) released into the bloodstream; EECS also use both hormones and fast-acting neurotransmitters to regulate activity of the gut-intrinsic ENS and the gut-extrinsic the parasympathetic vagus and sympathetic dorsal root neurons. These nerves provide a physical, fast-acting conduit that modifies activity across the CNS (Bohorquez et al., 2015; Bellono et al., 2017; Kaelberer and Bohorquez, 2018). Therefore, visceral stimuli influence not only visceral function via gut and brainstem reflexes but also homeostasis, reward, affect, and executive function (Kaelberer et al., 2020). While genes linked to ASD have been extensively studied for their roles in brain and behavior (Kozol et al., 2016), their function along the gut-brain axis has received much less attention. Underscoring the importance of visceral signals, recent studies in zebrafish have been able to accurately predict behavior sequences by integrating environmental stimuli and internal state (Johnson et al., 2020; Marques et al., 2020). Below we discuss the brain regions most relevant to the Gut-Brain axis; we also describe opportunities in zebrafish to better understand the gut-brain axis as it relates to symptoms in ASD.

For studies of the gut-brain axis, visceral sensory and motor pathways in diverse taxa are marked by their expression of the Paired-like homeodomain Phox2b transcription factor (Pattyn et al., 1999; Bertucci and Arendt, 2013; Nomaksteinsky et al., 2013; Harrison et al., 2014). When Phox2b function is disrupted in rodents, motor neurons that would normally innervate the viscera, find muscle targets, indicating that Phox2b functions to make specific CNS nuclei attend to the viscera (D’Autreaux et al., 2011). While this marker is conserved, one pronounced difference between mammals and zebrafish is that zebrafish and their relatives taste with sensory cells on their skin and lips and, as such, the first CNS relay for visceral sensations, the solitary tract nucleus (nTS), is lobed to accommodate expanded vagal, glossopharyngeal and facial inputs; nonetheless these lobes are thought to be functionally homologous to the gustatory portion of the nTS in mammals (Coppola et al., 2012). Work using tract tracing in zebrafish has helped to map the connectivity of largely conserved fish visceral brain circuits (Yanez et al., 2017).

## Setting the Stage

Even in advance of eating, sensations of external food stimuli as well as internal hunger or satiation states activate hypothalamic nuclei and play a highly conserved role in setting the stage (Sternson and Eiselt, 2017). Indeed hormonally regulated states of hunger, motivation to eat, satiety are similar in zebrafish and mammals (Jordi et al., 2015), though metabolic differences exist in leptin signaling associated with mammals being endotherms and fish being ectotherms (Gorissen and Flik, 2014). The ability to query the involvement of brain-wide circuits in zebrafish has been used to link behavioral states to brain activity (Randlett et al., 2015; Vanwalleghem et al., 2018). For example, seeing paramecia, a favored food of larval zebrafish, is sufficient to activate neural activity in the hypothalamus (Muto et al., 2017). Moreover, the transition between hunger and satiety can be mapped to activity in the ventromedial hypothalamus and lateral hypothalamus, respectively (Wee et al., 2019). In addition to hypothalamus, brainstem nuclei also respond to appetitive smell and taste in both fish and mammals (Vendrell-Llopis and Yaksi, 2015; Vincis and Fontanini, 2019) and sensorimotor integration during prey pursuit in zebrafish is modulated by feeding state (Filosa et al., 2016; Henriques et al., 2019). Due to the prevalence of eating disorders and sensory symptoms in ASD, an imbalance in sympathetic/parasympathetic tone is one of the hypotheses put forward to potentially explain these symptoms (Fenning et al., 2019). Currently, the physiological basis/es for eating difficulties in individuals with ASD is not well understood and is plagued by heterogeneity both in study design and how symptoms manifest across the spectrum (Margari et al., 2020). Recent studies show that while children with ASD are generally pickier about their food than neurotypical children (Babinska et al., 2020; Li C. et al., 2020), other symptoms may be unique to specific genetic forms of ASD. For example, in people with SYNGAP1 mutations, there is a correlation between eating and seizures (Vlaskamp et al., 2019).

## Gut-Brain Connectivity

Innervating the gut, parasympathetic and sympathetic neurons link directly to the CNS and convey information about digestive and microbiome status as well as mechanical and/or chemical insult (Browning and Travagli, 2014; Niu et al., 2020). Vagus and sympathetic nerves have both sensory/afferent and motor/efferent components that carry out visceral reflexes as well as integrating and conveying information to and from widespread brain regions across the CNS.

Most of the recent gut-brain axis literature has focused on the vagus nerve. The cell bodies of the motor component of the vagal neurons reside in dorsal motor nucleus (DMV) in the caudal brainstem. Motor innervation of the viscera is denser in the anterior GI tract (esophagus, stomach, and proximal small intestine) and activity in these organs tends to promote regulation of GI secretions and motility appropriate to the phase of digestion (Tache et al., 2006; Browning et al., 2017). In zebrafish, islet1:GFP transgenics label all the cranial motor neurons including the vagal motor nucleus (Higashijima et al., 2000). The DMV is functionally distributed from rostral to caudal, with the neurons innervating the viscera enriched caudally (Barsh et al., 2017; Isabella et al., 2020). Sensory vagus neuron cell bodies reside outside the CNS in the Nodose ganglion, therefore, it is relatively straight-forward to monitor neuronal activity in these cells to identify salient gut stimuli (Bai et al., 2019; Tsang et al., 2020; Zhang W. et al., 2020) and this approach that has recently been used in zebrafish (Ye et al., 2020). Work in rodents supports a critical role for an intact vagus nerve in the ability of *L. reuteri* bacteria to rescue social deficits in a *Shank3* ASD mouse model (Sgritta et al., 2019).

Sympathetic pre-ganglionic neurons when active during stress generally inhibit GI motility and secretion and also causes vasoconstriction that limits the blood supply to the viscera (Browning et al., 2017). Sensory sympathetic spinal afferents whose cell bodies reside in the dorsal root ganglia (DRG) also innervate the gut with denser innervation caudally (Muller et al., 2020). Sympathetic neurons are sensitive to digestion, injury, and microbes. While sympathetic innervation as it relates to digestion has not to our knowledge been studied in zebrafish, the DRG is accessible to electrophysiological recordings (Won et al., 2012) and both isl2b and ngn enhancers can drive expression of calcium sensors/light-gated channels in this cell type (Wright et al., 2010; Stil and Drapeau, 2016; Hall and Tropepe, 2018). Using these tools in zebrafish larvae could help elucidate GI stimuli and insults that activate sympathetic spinal afferents *in vivo* and how this activity is impacted zebrafish ASD models.

The brainstem/medulla oblongata is rich in nuclei that receive, process, and respond to sensory information from the GI tract. Vagal inputs directly innervate the Area Postrema (AP) and the Nucleus of the Solitary Tract (nTS) (Ma, 1997; Kaslin and Panula, 2001; McLean and Fetcho, 2004; Coppola et al., 2012). The AP is one of the few areas of the CNS that is not protected by the blood brain barrier and as such is responsive to factors/toxins in the bloodstream; neuronal activity in the AP is linked to the symptom of nausea (Zhang C. et al., 2020). Consistent with functions established in mammals, the zebrafish AP has been shown to be responsive to the pain-inducing Trp1A agonist AITC (Haney et al., 2020). The nTS serves as the first CNS relay to many other brain regions (Coppola et al., 2012; Yanez et al., 2017; Han et al., 2018) including the secondary gustatory nucleus aka parabrachial nucleus (PBN) as well as the DMV. Both nTS and PBN are marked by Phox2b expression with transgenic drivers available (Nechiporuk et al., 2007; Coppola et al., 2012). The zebrafish nTS is dorsal and sheetlike and, as such, is amenable to *in vivo* imaging studies (Vendrell-Llopis and Yaksi, 2015). Taste and visceral inputs map to different regions of the nTS in mammals (Vincis and Fontanini, 2019; Kaelberer et al., 2020) and in fishes, including zebrafish, visceral inputs map to the caudal part of the nTS in the adult brain (Kermen et al., 2013; Yanez et al., 2017). As a nucleus that integrates direct inputs from both viscera and CNS, the nTS holds promise for elucidating what aspects of the gut-microbiome-brain signaling may be altered in zebrafish ASD models.

## Gut Feelings

In addition to visceral reflexes mediated at the level of hypothalamus and brainstem, widespread CNS nuclei mediating memory, emotion/affect, and motivation have been shown in rodents to also be responsive to gut stimuli (Kaelberer et al., 2020). Severing vagal afferents results in increased exploratory behaviors and risk-taking, heightened auditory-based fear conditioning, and altered neurotransmitters in the limbic system (Klarer et al., 2014, 2018). Stimulation of vagal afferents entering the brainstem on the right side engage a PBN to nucleus accumbens to dorsal striatum reward pathway and stimulating this pathway sufficient to elicit behaviors consistent with reward (Han et al., 2018). Another pathway activated through the vagus is the nTS to medial septum to hippocampus that when disrupted interferes with spatial memory (Suarez et al., 2018). Analogous zebrafish brain regions to those mediating memory, emotion/affect, and motivation in mammals are continuing to be elucidated in zebrafish, and anatomical studies indicate similar connectivity between visceral circuits and these brain regions in zebrafish (Yanez et al., 2017).

Elucidating the link between GI distress and negative behavioral symptoms could improve symptom management. Not only are GI symptoms common in ASD, but they correlate with more pronounced irritability, social withdrawal, stereotypy, hyperactivity, and sleep disturbances (De Rubeis and Buxbaum, 2015; McCue et al., 2017; Penzol et al., 2019). Such an intimate link between gut and brain symptoms is well-established in Parkinson’s disease where constipation often precedes the motor disturbances (Fasano et al., 2015; Mayer et al., 2015; Liddle, 2018; Ramprasad et al., 2018). The possibility that gut symptoms could contribute to the development of neurological symptoms has also been suggested in ASD (Mayer et al., 2014; Eshraghi et al., 2018) with recent studies providing empirical support (Sgritta et al., 2019).

## Zebrafish as a Model for Microbial Studies and Their Potential Role in ASD

Humans are extensively colonized with microbial species, resulting in several distinct microbiomes determined by geographic distribution across the host’s body (Human Microbiome Project Consortium, 2012). Interactions between host and microbiome are complex, and our understanding of the bi-directional influence between the two is evolving as the scientific community adopts a less human-centric view (reviewed in Wiles and Guillemin, 2020). Nonetheless, the microbiome has long been understood to play an important role in host form and function. While the GI microbiome was traditionally thought to predominately interact with its host through nutrient processing, further study has shown that it is capable of influencing the host immune system and nervous system as well, and thus has direct implications in disorders like ASD.

The composition of the human GI microbiome is largely determined by functional, rather than taxonomic, qualification. The gut microbiome does not need any particular species profile to operate and maintain a commensal relationship with the host. Rather, it requires certain functions to be performed, which can be executed by any number of potential microbial species. In humans, the microbiome is composed of predominantly Firmicutes and Bacteroidetes species (Human Microbiome Project Consortium, 2012). Much like an ecosystem of macroorganisms, the human GI microbiome is influenced by its physical habitat (gut morphology), resource availability (host diet), and interspecies interaction (both between microorganisms and between the microbiome and host itself). Likewise, both the development and the function of the vertebrate immune and nervous system are affected by the GI microbiome. The gut microbiome is capable of exerting an effect on neurological functioning through several pathways, with metabolites able to travel through the host bloodstream or act locally upon the vagus (Schroeder and Backhed, 2016; Fulling et al., 2019). Immune responses elicited by microbes or their metabolites can also have implications on the brain and its function.

The GI microbiome also plays an important role in the development of the host immune system. Early-colonizing microbial species provide “training” to immune effectors, allowing them to become accustomed to commensal communities and to distinguish between them and pathogenic microorganisms. Severe negative consequences can occur when this process is interrupted or prevented. In germ-free mice, colonic epithelial cells are incapable of raising an immune response upon exposure to a bacterial pathogen (Lundin et al., 2008). Similar dysfunction can be seen in germ-free zebrafish, which display impaired differentiation of GI cell types such as goblet and enteroendocrine cells along with impaired nutrient uptake and death prior to adulthood if not conventionalized (Bates et al., 2006; Melancon et al., 2017). Human infants that avoid exposure to maternal microbiomes through cesarean delivery and/or formula feeding display increased inflammatory responses and autoimmune disorders (Toscano et al., 2017; Koch et al., 2018). As the microbiome shapes the immune system, the innate immune system of the host in turn shapes the native microbiome to one tailored to the individual’s metabolic needs (Thaiss et al., 2016).

The microbiome has been shown to be capable of affecting or inducing multiple aberrant neurological phenotypes in various study systems. Some of these alterations have been traced to bacterial metabolites such as short chain fatty acids (SCFAs). Elevated levels of SCFAs have been directly detected in fecal samples from autistic patients (Wang et al., 2012). Although it was undetermined if those levels were mediated by the gut microbiome, other studies have likewise shown increased numbers of the Clostridia family (enterobacteria that are key producers of various SCFAs) in stool samples from individuals with autism. Studies in rats have recapitulated a behavioral phenotype resembling that of autistic patients by treatment with propionic acid, a short-chain fatty acid produced by Clostridia (MacFabe et al., 2007). Similar associations have been found in models of Parkinson’s disease, where bacterial SCFAs were sufficient to promote neuroinflammation in the mouse subjects (Sampson et al., 2016). The microbiome has also been shown to influence neurological conditions through modulation of the immune system (Benakis et al., 2016; Schroeder and Backhed, 2016). Microbial attenuation of inflammatory cytokines has been linked with reductions in anxiety (Cryan and O’Mahony, 2011) and antibiotic treatment in a stroke mouse model has been shown to confer neuroprotection post-ischemic injury through a reduction in intestinal immune effectors (Benakis et al., 2016). In addition to influencing immune activity, microbes in the gut are capable of altering hormone signaling as well. Spore-forming bacterial species in the gut have been shown to induce serotonin production by enterochromaffin cells through the release of several metabolites. This promotion of serotonin production was found to ameliorate the reduced GI motility seen in germ-free mice when the subjects were colonized with the spore-forming species (Yano et al., 2015).

Zebrafish are powerful model organisms for experiments involving microbiome contribution. As they are initially colonized by microbes via their environment, it is possible to raise them in sterilized conditions that result in germ-free individuals (Rawls et al., 2004). Once germ-free subjects are generated, experiments involving selective colonization, introduction of metabolites, and conventionalization effects are possible. The zebrafish gut is able to be imaged *in vivo* in the early life stages due to the optical transparency of larvae. This allows for examination of gut function like motility, and barrier function (Marjoram et al., 2015), as well as location and interspecies dynamics of fluorescently labeled bacterial species (Wiles and Guillemin, 2020). The gut is also easily dissectible, allowing for extraction of the microbiome for 16S sequencing.

## Zebrafish as a Model for GI Form and Function, and Modeling ASD-Related GI Dysfunction

The use of zebrafish models for GI research has increased considerably over the last three decades (Figure 1), with publications exploring conserved and unique aspects of GI form and function, as well as more nuanced topics including specific disease models (reviewed in Zhao and Pack, 2017) and external impacts like microplastic exposure (Qiao et al., 2019) and chemotherapy treatment (Van Sebille et al., 2019). Like the animal model as a whole, the zebrafish GI system has many physiological similarities and differences with its mammalian model counterparts. There is conservation of key GI cell types in the zebrafish GI tract, with absorptive enterocytes, mucus producing goblet cells, chemo/mechano-sensitive enteroendocrine cells, and the innervation by the ENS (Roy-Carson et al., 2017) filling similar functional niches. The digestive tract and its accessory organs similarly form from a strip of endodermal tissue in early development (as early as 21 hpf in zebrafish) (Wallace and Pack, 2003). While there is some debate as to whether these organs develop individually or from the same interconnected endodermal strip (Ng et al., 2005), the differential expression of conserved genes key to GI development begins as early as 18hpf. Expression of these orthologs, such as pharyngeal endoderm associated gene *axial*, liver development gene *hhex*, pancreas development gene *pdx*, and esophageal gene *gata-6*, suggest that GI development in zebrafish differs slightly from mammals, with progenitors for the liver, pancreas, and pharynx existing before morphogenesis of the GI tract itself is complete. Similarly, small differences exist for the roles of morphogens like sonic-hedgehog (*shh*); required as a negative regulator for pancreatic development in mammals, in zebrafish it appears to be a positive regulator (Wallace and Pack, 2003). Like in mammals, the zebrafish GI tract has distinct functional layers, with an epithelial mucosal layer, and an underlying muscular layer innervated by the ENS. Unlike mammals, however, zebrafish lack a submucosal layer, villi are replaced by broad folds in the mucosal layer, the ENS is not organized into ganglia (acting instead as a nerve net), and the proliferative crypts of Lieberkuhn are absent (though proliferating cells still expand from stem cell niches at the base of the mucosal folds) (Wallace and Pack, 2003; Ng et al., 2005; Wallace et al., 2005; Uyttebroek et al., 2010). From a functional perspective, the zebrafish also has other simplifications when compared to mammalian models; they lack a true stomach (separated by sphincters and containing acid-producing Paneth cells) and instead have an intestinal bulb. This bulb likely acts as a reservoir for food, and motility patterns in this region are both anterograde and retrograde, acting to mix and break food down mechanically (Holmberg et al., 2004). These and other differences point to an overall simplification of the GI tract in zebrafish. Although components of the GI tract (including development, cell types, and molecular signaling) are conserved between zebrafish and mammals, there are important differences that need to be acknowledged when using zebrafish as a model for GI research.

From a GI-research prospective, zebrafish offer a key advantage over mammalian models: their external fertilization, development, and early transparency make studying GI function *in vivo* significantly easier. Measurements of digestive transit, peristaltic rate, and general ontogeny of GI motility can be made before the larvae begin feeding (with spontaneous motility developing before 5 dpf) (Holmberg et al., 2004), and do not require any complex or potentially variable-confounding surgical procedures. The measurement of motility in zebrafish also has its disadvantages; since this is a relatively new branch of zebrafish research, the approaches have not been standardized, with multiple labs (including our own) creating their own software for measurements of motility and transit (Field et al., 2009; Rich, 2009; Jordi et al., 2015; James et al., 2019). Although these different models share similar components and aims, the differences could present possible complications when comparing results. For instance, in our own model, while measuring transit and motility are relatively straightforward, determining the force of muscular contraction is difficult, and potentially confounding variables (such as food particles in the GI tract) mean that measuring GI motility specifically (and excluding the movement of particulates) becomes difficult. Additionally, as most motility software was developed for in-house use, the user-interface and technical aspects of each model present possible speedbumps for researchers unfamiliar with the software. The field, as a whole, would benefit from a unified method for GI motility measurement, especially when attempting to tackle questions on ASD-related GI distress.

While there is increasing interest in zebrafish-based GI research, and similarly, increasing interest in the relationship between ASD and GI dysfunction in non-zebrafish models (Figure 1), there has not been a coupling of the two. In fact, to our knowledge and excluding our own work, there have only been three publications that include data on ASD-related GI symptoms using zebrafish models, one of which is technically not ASD-specific (focusing on CHARGE syndrome, which has overlap with ASD but is distinct) (Bernier et al., 2014; van der Vaart et al., 2017; Cloney et al., 2018). This represents, in our view, a significant shortcoming that should be addressed in future studies. Previous work from our lab (James et al., 2019) attempts to address this shortcoming, and utilizes some of the aforementioned zebrafish GI assays to look at GI dysfunction in a monogenic ASD model. In this work, we focus on GI dysmotility found in CRISPR mutant of ASD related gene *shank3ab.* We found that while there was no difference in enteric neuron count, there was a significant decrease in the expression of serotonin-positive enteroendocrine cells in *shank3ab* mutants. We also note that RNA-seq data from a collaborating lab has found that *shank3ab* expression is detected in this population of cells (Wen et al., 2020). Interestingly, recent work in mouse models has found that enteroendocrine cells (specifically serotonin producing enterochromaffin cells) synapse directly with the ENS (Bellono et al., 2017). Taken together, this suggests that changes in GI physiology can have profound impacts on GI-CNS communication, and that there may be a non-CNS role for genes like *shank3ab*, which are largely understood and studied through their context in the CNS. To add further complexity to the picture, we also found increased goblet cell populations when comparing adult WT and *shank3ab* mutant fish, a difference not present in larval fish and indicative of possible age-related GI inflammation; we are currently exploring the microbial implications behind this finding. This work, coupled with the previously mentioned ASD-GI publications presents possible venues for establishing ASD-related GI pathophysiology, and it sets a foundation for future studies.

## Discussion: Drawing Conclusion and Looking Forward

In this review, we have discussed some of the important research in the gut-brain-microbiome field, as well as presented the zebrafish as an ideal model for future multidisciplinary ASD studies. Moving forward, we hope research in zebrafish models increasingly integrates behavioral phenotypes and comorbidities of sleep, seizure, and GI function to obtain a more complete understanding of how genetic changes that can cause ASD impact the organism as a whole. To this end, there have been recent advances in the development of social assays that have helped characterize the neural circuits involved in social behavior (Dreosti et al., 2015; Stednitz et al., 2018). Additionally, adult social behaviors in a zebrafish ASD model have reduced shoaling behavior (Liu et al., 2018). While the bulk of current zebrafish ASD models are caused by indels that result in premature stop codons, recently developed CRISPR/Cas9 technologies in zebrafish are making tissue-specific mutagenesis and knockins that replicate patient-specific mis-sense mutations more broadly feasible (Albadri et al., 2017; Li J. et al., 2020). These technological advances continue to make the zebrafish an increasingly valuable model for ASD research.

It is also important to briefly highlight the role zebrafish are playing in high-throughput drug screening and drug discovery for treating ASD comorbidities. The zebrafish is becoming a well-established model for drug screening (Haesemeyer and Schier, 2015; Hoffman et al., 2016; Cassar et al., 2020), and is a particularly powerful model for high-throughput screens aimed at tailoring integrative care on a patient-symptom basis. The broad heterogeneity within ASD warrants an equally broad scope of research on different palliative drugs. It should be noted, however, that this heterogeneity also complicates the treatment of comorbidities along with the core behavioral phenotypes associated with ASD. Many current drugs are aimed at treating behavioral problems such as irritability and aggression (Stachnik and Gabay, 2010; Coleman et al., 2019), and in doing so frequently overlook downstream side-effects that might also be contributing to the initial behavioral issue. Risperidone, for example, is often used to treat problematic behavioral issues associated with ASD by inhibiting dopaminergic D2 receptors and serotonergic 5-HT2A receptors and can lead to constipation in human patients, which can in turn lead to a worsening of non-verbal behaviors such as agitation and anxiety. Similarly, common GI medications used to treat delays in gut transit (such as Metoclopramide) can lead to changes in behavior in zebrafish due to a compensatory mechanism following weakened dopamine signaling (Shontz et al., 2018). To further complicate the issue, the relationship between the GI microbiome and drug application is not well understood. As such, we believe the zebrafish also serves as an ideal model for ASD drug screening, not only for behavioral impacts, but for GI and microbiome impacts as well (Cassar et al., 2015). As their power in translational ASD research is already well established (Ijaz and Hoffman, 2016; Sakai et al., 2018), zebrafish could serve as an intersection between patient care and foundational exploratory research, with pre-clinical trials of newly discovered drugs helping to inform which treatments have the best benefit for multiple symptoms.

Autism spectrum disorder research has largely been compartmentalized, whether into behavioral, molecular, or microbial aspects. Given the interconnected regulatory pathways and feedback loops existing between the CNS, GI tract, and microbiome, we believe this compartmentalization acts to the detriment of a broader understanding of the disorder. Consequently, if the current lack of ASD-related GI research is filled, it could stand to provide critical components to both CNS and microbiome research, while also producing important foundational information on potential ASD-related GI pathophysiology.

## Author Contributions

All authors contributed to the research, writing, and editing of this review. DJ and JD conceived the scope of the review, coordinated efforts among authors, and wrote the gastrointestinal and CNS sections. ED wrote the bulk of the microbiome section. JY wrote the bulk of the drug screening section. BM aided in research and references to clinical studies.

## Conflict of Interest

The authors declare that the research was conducted in the absence of any commercial or financial relationships that could be construed as a potential conflict of interest.
